# Modulation of autoimmune diabetes by N-ethyl-N-nitrosourea- induced mutations in non-obese diabetic mice

**DOI:** 10.1242/dmm.049484

**Published:** 2022-06-01

**Authors:** Lucienne Chatenoud, Cindy Marquet, Fabrice Valette, Lindsay Scott, Jiexia Quan, Chun Hui Bu, Sara Hildebrand, Eva Marie Y. Moresco, Jean-François Bach, Bruce Beutler

**Affiliations:** 1 Université Paris Cité, Institut Necker Enfants Malades, F-75015 Paris, France; 2 INSERM UMR-S1151, CNRS UMR-S8253, Institut Necker Enfants Malades, F-75015 Paris, France; 3 Center for the Genetics of Host Defense, University of Texas Southwestern Medical Center, Dallas, TX 75390, USA

**Keywords:** Non-obese diabetic (NOD) mice, Autoimmune diabetes, Type 1 diabetes, Genetics, Risk locus, Protective locus, Automated meiotic mapping

## Abstract

Genetic association studies of type 1 diabetes (T1D) in humans, and in congenic non-obese diabetic (NOD) mice harboring DNA segments from T1D-resistant mice, face the challenge of assigning causation to specific gene variants among many within loci that affect disease risk. Here, we created random germline mutations in NOD/Nck^H^ mice and used automated meiotic mapping to identify mutations modifying T1D incidence and age of onset. In contrast with association studies in humans or congenic NOD mice, we analyzed a relatively small number of genetic changes in each pedigree, permitting implication of specific mutations as causative. Among 844 mice from 14 pedigrees bearing 594 coding/splicing changes, we identified seven mutations that accelerated T1D development, and five that delayed or suppressed T1D. Eleven mutations affected genes not previously known to influence T1D (*Xpnpep1*, *Herc1*, *Srrm2*, *Rapgef1*, *Ppl*, *Zfp583*, *Aldh1l1*, *Col6a1*, *Ccdc13*, *Cd200r1*, *Atrnl1*). A suppressor mutation in *Coro1a* validated the screen. Mutagenesis coupled with automated meiotic mapping can detect genes in which allelic variation influences T1D susceptibility in NOD mice. Variation of some of the orthologous/paralogous genes may influence T1D susceptibility in humans.

## Abstract (precis)

**Summary:** Automated meiotic mapping, which allows rapid discovery of induced germline disease modifier mutations, reveals influential loci in mice with an autoimmune diabetes phenotype, identifying genes that may influence type 1 diabetes susceptibility in humans.

## INTRODUCTION

Type 1 diabetes (T1D), also termed insulin-dependent diabetes or juvenile diabetes, is a polygenic autoimmune disease in which autoreactive T cells destroy insulin-producing β cells of the islets of Langerhans. Some 50-80% of risk for T1D is heritable and the genetic factors that drive T1D have been intensively pursued. Prior to genome-wide association studies (GWAS), a classical gene candidate approach was used to first identify the human leukocyte antigen (HLA) association (Nerup et al., 1974; Polychronakos and Li, 2011); this association remains the strongest by far, with reported odds ratios ranging from 0.02 to >11 for specific DR-DQ haplotypes (Erlich et al., 2008). Outside the HLA, two other confirmed candidates were the insulin variable number of tandem repeats locus (INS-VNTR; odds ratio=2.4) (Barratt et al., 2004; Bell et al., 1984) and the *CTLA4* gene (Nisticò et al., 1996; Ueda et al., 2003), which together contribute about 15% of the risk. Then Bottini et al. reported that a single-nucleotide polymorphism (SNP) in *PTPN22*, encoding protein tyrosine phosphatase non-receptor type 22 (also known as lymphoid protein tyrosine phosphatase, LYP), a suppressor of T-cell activation, was also associated with T1D (odds ratio >1.5) (Bottini et al., 2004). The *IL2RA* gene, encoding interleukin 2 receptor subunit alpha, has consistently been reported to have an odds ratio >1.5 (Vella et al., 2005). GWAS have uncovered approximately 100 genomic regions associated with T1D risk (Pociot, 2017; Robertson et al., 2021), including many containing cis-acting gene regulatory sequences (Onengut-Gumuscu et al., 2015; Kim et al., 2021). The creation of congenic non-obese diabetic (NOD) mouse strains by limited introgression of DNA from T1D-resistant strains (C57BL/6J, C57BL/10J and NZW) into NOD strains through outcrossing and repeated backcrossing has permitted quantitative trait locus (QTL) mapping of at least 50 insulin-dependent diabetes (Idd) loci that can suppress or augment the development of T1D (Driver et al., 2012; Ridgway et al., 2008; Garchon et al., 1991; Luan et al., 1996). These approaches revealed a substantial contribution (up to ∼50%) of certain HLA class II or major histocompatibility complex (MHC) class II alleles to T1D disease risk in humans and NOD mice, respectively (Todd et al., 1987; Svejgaard et al., 1983; Hattori et al., 1986; Erlich et al., 2008). Outside of the HLA/MHC loci, risk variants have modest to low effect sizes (Pociot, 2017).

Despite clear evidence of genetic predisposition to T1D, the preponderance of this risk remains unascribed to individual genes (Pociot, 2017). Difficulty in resolving causative variants and attributing causation to specific genes is due in part to the abundance of genetic variation involved. Many risk loci may be present in a given study population and each risk locus found by GWAS, or by QTL mapping in congenic mouse strains, contains numerous genes, and each gene within the locus may have one or more non-synonymous coding/splicing differences. Rather than coding changes, non-coding variants within a locus may instead be responsible for altering the expression of gene(s) thousands of nucleotides distant (Ram et al., 2016). Usually, the process of gene identification relies on prior non-genetic data on candidate gene expression, localization and function, when available; thus, the critical changes within each locus that are directly responsible for modification of phenotype are often speculative. Although the NOD congenic strain approach and GWAS of T1D were initiated respectively in the mid-1980s and 2000s, causative genes remain unresolved for most Idd loci and T1D GWAS loci, illustrating the enduring challenge of causative gene identification within risk loci or QTLs (Chen et al., 2018; Pociot, 2017).

Substrains of NOD mice derived from the inbred strain NOD/Shi, first reported in 1980 by Makino et al. in Japan (Makino et al., 1980), have been bred all over the world. Our colony at Hôpital Necker in Paris (NOD/Nck) was started in 1986 (Hunger et al., 1996). As previously described, we established by brother-sister breeding two NOD sublines with low T1D incidence (NOD/Nck^L^) and high T1D incidence (NOD/Nck^H^), presumably caused by variation arising from recent genetic drift (Foray et al., 2021). We used automated meiotic mapping (AMM) (Wang et al., 2015) to attribute the high T1D incidence in NOD/Nck^H^ (70%) versus NOD/Nck^L^ (20%) male mice to a recessive missense mutation of *Dusp10* (Foray et al., 2021). Here, we accelerated the generation of variants impacting T1D susceptibility by mutagenizing mice with the chemical mutagen N-ethyl-N-nitrosourea (ENU), and then used AMM to identify *de novo* modulators of autoimmune diabetes. With the primary goal of identifying suppressor mutations that reduce the incidence of T1D, we elected to mutagenize and screen mice on the high-incidence NOD/Nck^H^ background, on which approximately 80% and 70% of female and male mice develop T1D, respectively (Foray et al., 2021). By introducing a relatively small number of genetic changes by ENU, this approach circumvents the technical challenges of pinpointing causative genes within QTLs as in NOD congenic mice. We identified several mutations among 553 induced at random that appear to accelerate, delay or prevent the development of T1D.

## RESULTS

**Fig. 1.**
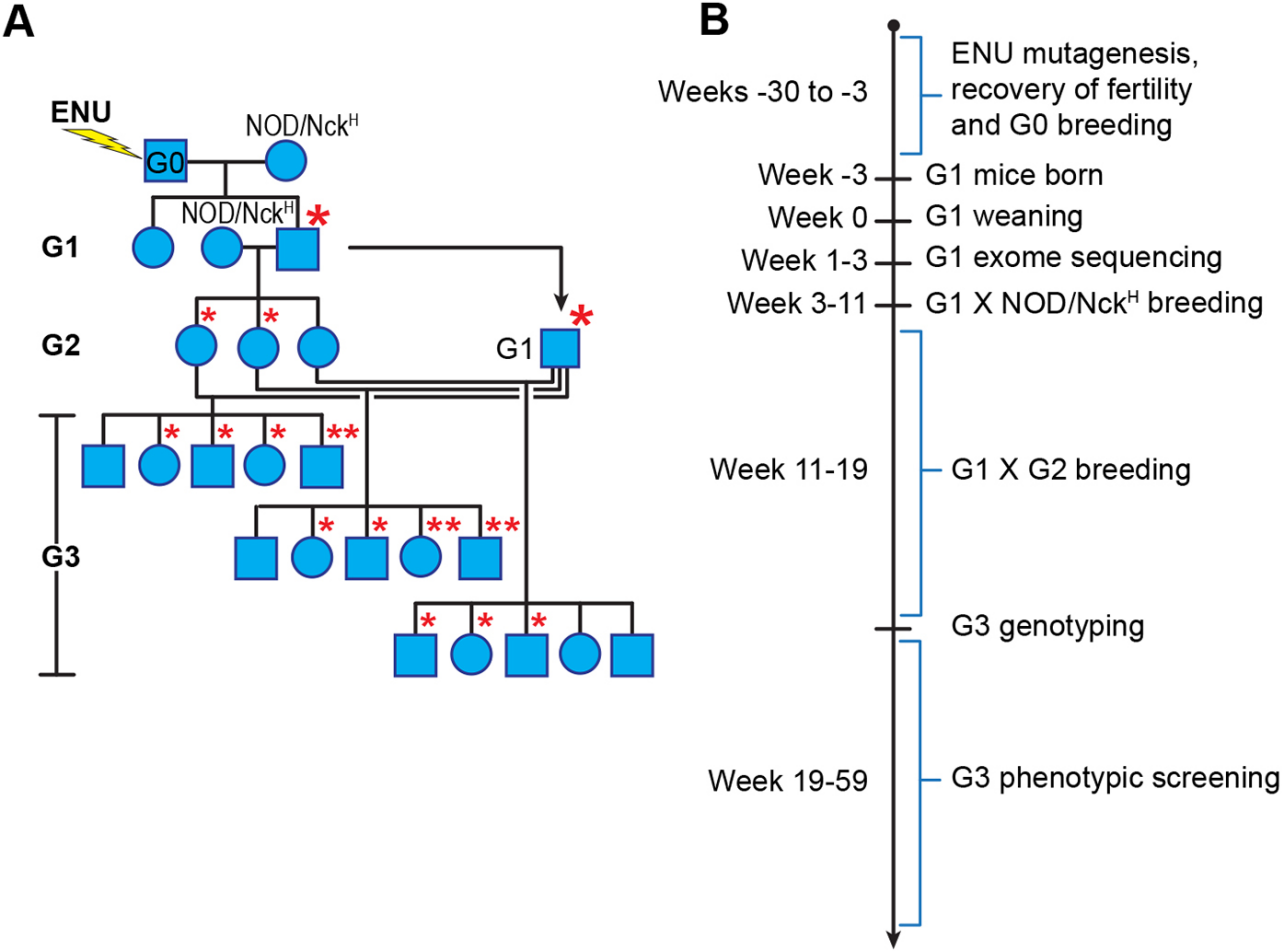
**Generation of NOD/Nck^H^ G3 mice for phenotypic screening.** (A) Breeding plan to produce G3 mice. NOD/Nck^H^ males were mutagenized with ENU (G0) and bred with NOD/Nck^H^ females. NOD/Nck^H^ female mating partners for G1 males were of the same generation as those bred to G0 males. Asterisks represent mutations originating from the G0 male; larger asterisks indicate initial germline transmission of the mutation. (B) Timeline for the production of G3 mice for screening.

Because different mouse strains have different sensitivities to the chemical ENU (Davis et al., 1999) and no published data are available on optimal dosage of ENU for NOD mice, we tested doses of 100 mg ENU/kg body weight and 125 mg ENU/kg body weight, administered intraperitoneally three times at weekly intervals (Georgel et al., 2008; Stottmann and Beier, 2014; Davis et al., 1999). Eighteen NOD/Nck^H^ male mice were mutagenized, nine each at the lower and higher doses. Twenty-one NOD/Nck^L^ male mice were also mutagenized for the purpose of examining spermatogenesis (Fig. S1A) (ten at the lower dose and 11 at the higher dose). The impacts on sperm counts of the 100 mg/kg and 125 mg/kg doses were similar over a period of 9 weeks after the third ENU injection (Fig. S1B). We monitored survival and return of fertility in mutagenized NOD/Nck^H^ mice over a period of 8 weeks post-ENU. Among the nine NOD/Nck^H^ mice treated with the higher dose, three died by 8 weeks post-ENU, and the remaining six mice failed to regain fertility by 8 weeks post-ENU and were sacrificed. At the lower dose, all nine NOD/Nck^H^ mice survived to at least 13 weeks post-ENU and three regained fertility at 18 weeks post-ENU. Fertile mutagenized mice (designated G0) were mated to one to three NOD/Nck^H^ female mice, which gave birth to 24 G1 male mice in total. Further breeding (Fig. 1A) finally resulted in 633 female G3 mice within 14 pedigrees (Table 1), carrying an average of 42.4 ENU-induced coding or splice site mutations per pedigree (594 total mutations identified by exome sequencing of the G1 founders). Ten pedigrees contained ≤14 female G3 mice per pedigree and were not screened. The timeline to produce a pedigree to the G3 generation is shown in Fig. 1B.

**Table 1.**
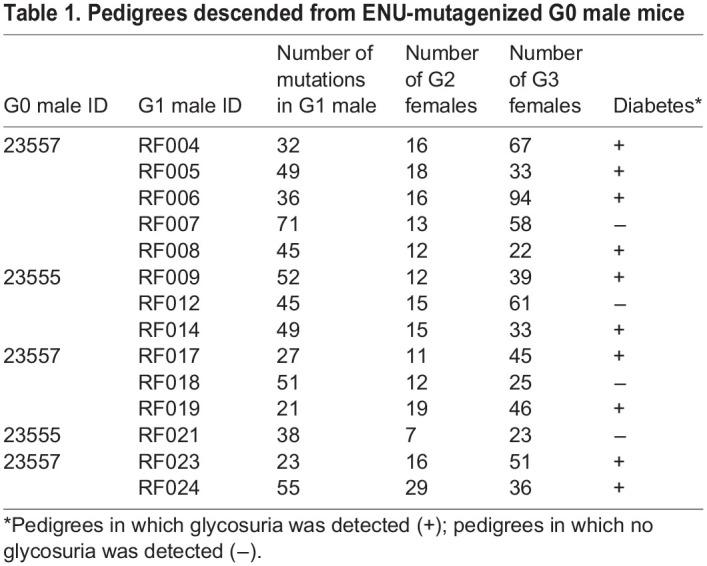
Pedigrees descended from ENU-mutagenized G0 male mice

**Fig. 2.**
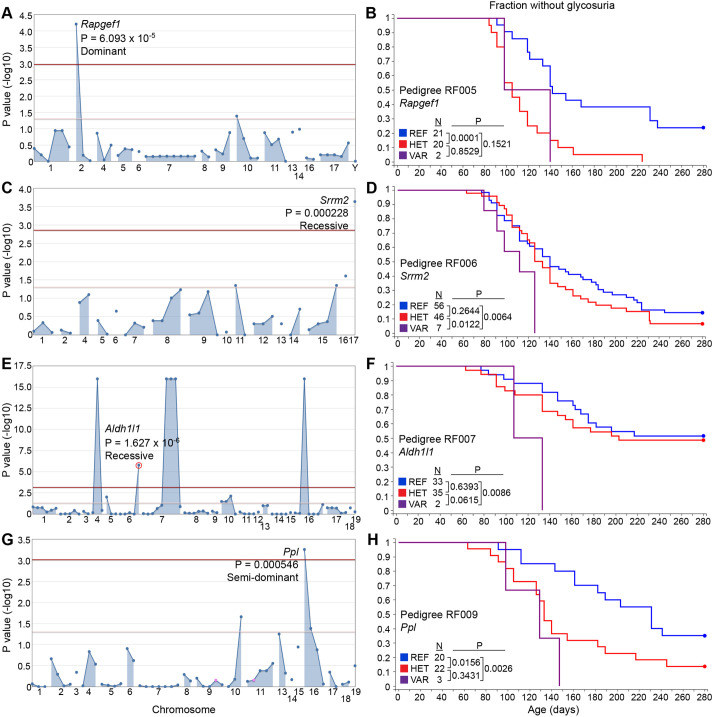
**Accelerated T1D phenotypes.** (A,C,E,G) Manhattan plots. *P*-values for non-linkage (−log_10_) were plotted versus the chromosomal positions of mutations in each pedigree. Red and pink lines represent thresholds for *P*=0.05 with or without Bonferroni correction, respectively. In each plot, a single peak is labeled with the mutated gene's symbol, *P*-value and inheritance model used for *P*-value calculations. (B,D,F,H) Kaplan–Meier plots showing glycosuria onset age for G2 and G3 mice with or without the mutation in the corresponding Manhattan plot to the left. *P*-values were calculated using the logrank test. HET, heterozygous for the mutant allele and the reference allele; REF, homozygous for the reference allele; VAR, homozygous for the mutant allele.

**Fig. 3.**
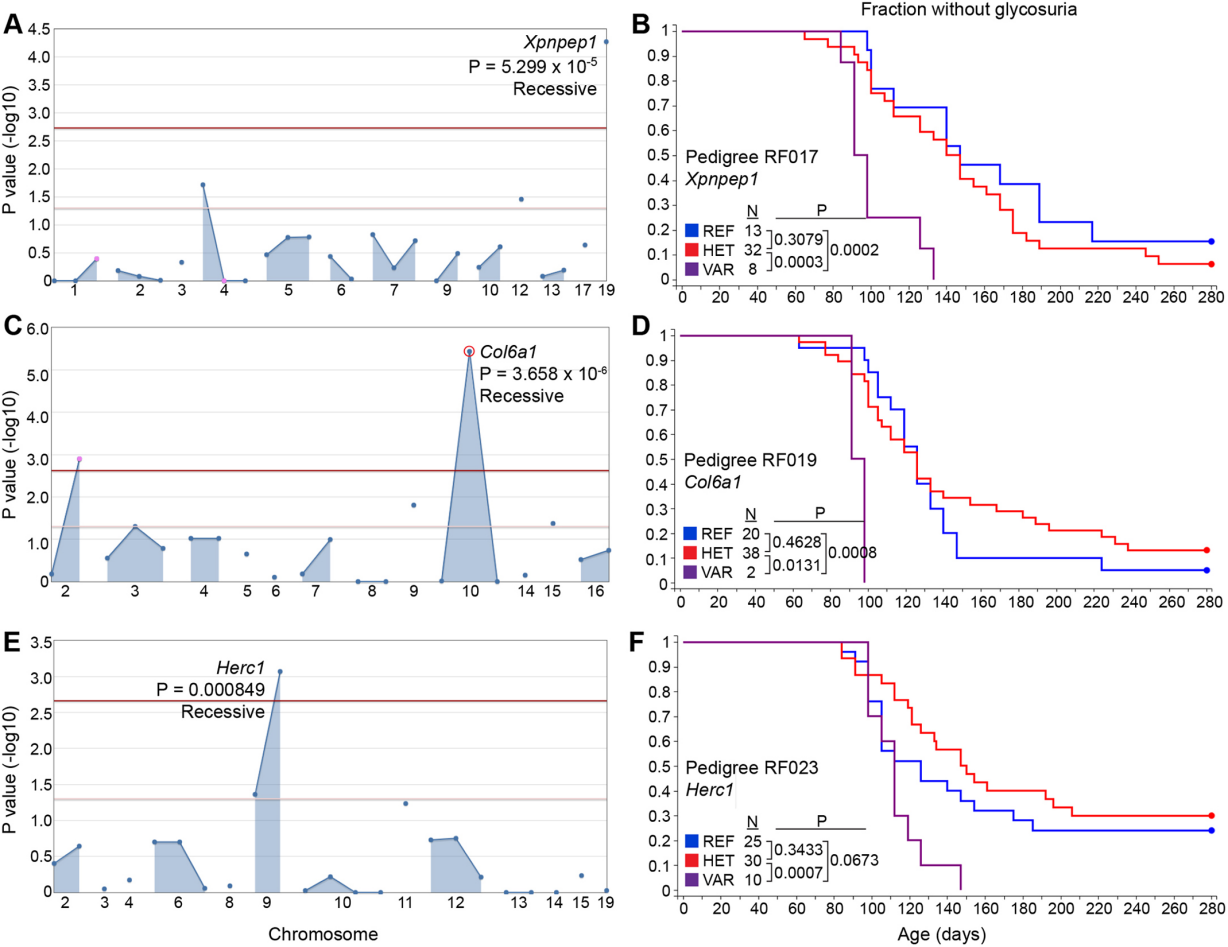
**Accelerated T1D phenotypes.** (A,C,E) Manhattan plots. *P*-values for non-linkage (−log_10_) were plotted versus the chromosomal positions of mutations in each pedigree. Red and pink lines represent thresholds for *P*=0.05 with or without Bonferroni correction, respectively. In each plot, a single peak is labeled with the mutated gene's symbol, *P*-value and inheritance model used for *P*-value calculations. (B,D,F) Kaplan–Meier plots showing glycosuria onset age for G2 and G3 mice with or without the mutation in the corresponding Manhattan plot to the left. *P*-values were calculated using the logrank test. HET, heterozygous for the mutant allele and the reference allele; REF, homozygous for the reference allele; VAR, homozygous for the mutant allele.

**Fig. 4.**
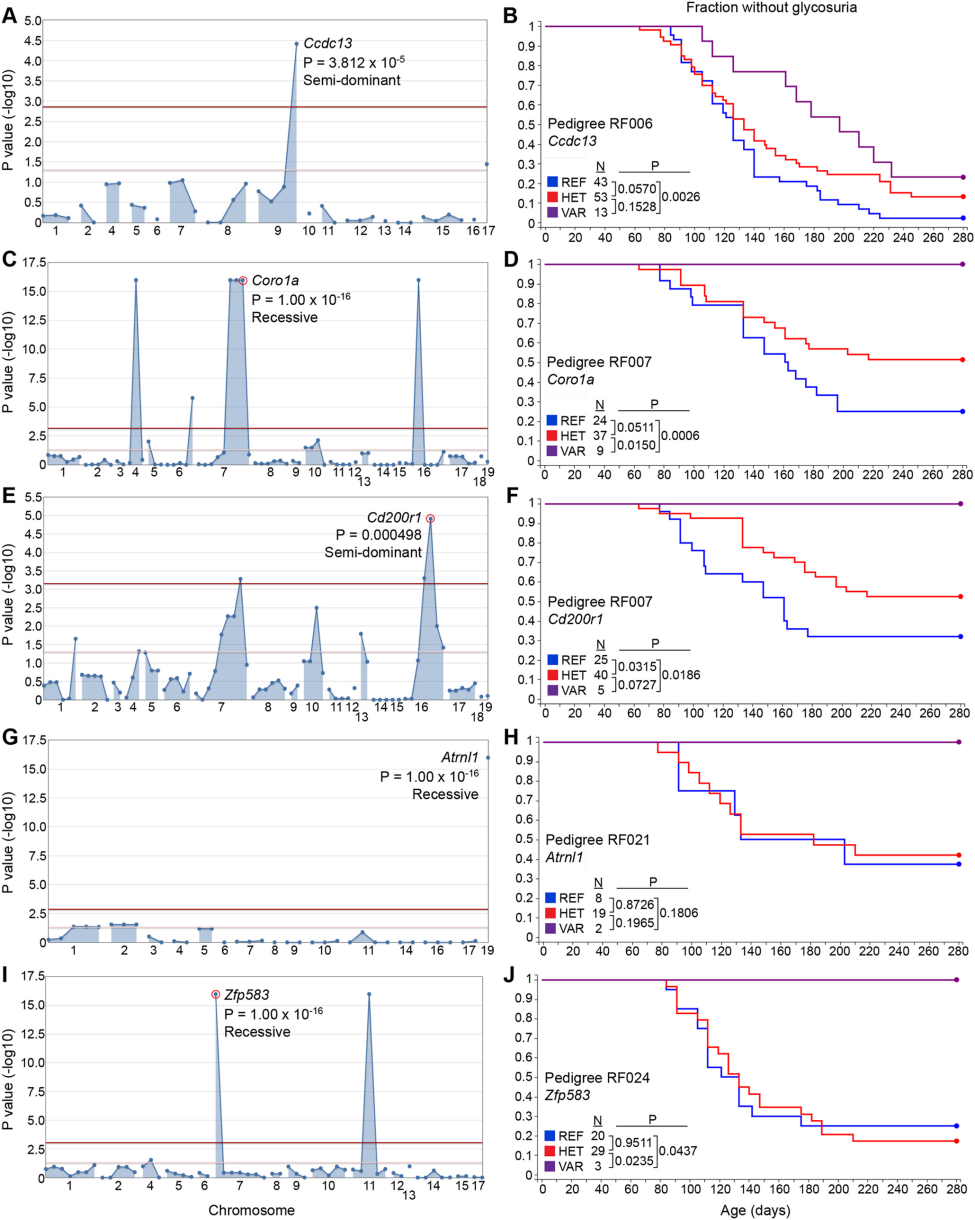
**Delayed T1D phenotypes.** (A,C,E,G,I) Manhattan plots. *P*-values for non-linkage (−log_10_) were plotted versus the chromosomal positions of mutations in each pedigree. Red and pink lines represent thresholds for *P*=0.05 with or without Bonferroni correction, respectively. In each plot, a single peak is labeled with the mutated gene's symbol, *P*-value and inheritance model used for *P*-value calculations. (B,D,F,H,J) Kaplan–Meier plots showing glycosuria onset age for G2 and G3 mice with or without the mutation in the corresponding Manhattan plot to the left. *P*-values were calculated using the logrank test. HET, heterozygous for the mutant allele and the reference allele; REF, homozygous for the reference allele; VAR, homozygous for the mutant allele.

G2 and G3 female mice (*n*=844) were used for phenotypic screening in which they were monitored for glycosuria weekly through 40 weeks of age. A cohort of untreated NOD/Nck^H^ females from the same generation as those treated with ENU was monitored in parallel as ‘wild-type’ (WT) controls. Genome saturation was assessed at 0.35% of annotated autosomal protein-encoding genes modified by damaging and/or null alleles screened twice or more in the homozygous state (Wang et al., 2018). We performed single locus linkage analysis using AMM to map the age of T1D onset as a quantitative trait based on Kaplan–Meier analysis in each of the 14 pedigrees (Wang et al., 2015). *P*-values for non-linkage of mutations to altered age of T1D onset were calculated using recessive, dominant and semi-dominant models of inheritance (Wang et al., 2015). We called candidate mutations meeting the following criteria: (1) present in pedigrees with at least 20 G3 mice, (2) screened in at least two homozygous mutant mice, (3) the reference allele was also screened in the homozygous state in at least two mice, and (4) *P*<0.05 with Bonferroni correction. After applying these criteria, twelve mutations in twelve genes stood as candidates that altered the age of onset of T1D relative to the non-mutagenized parental NOD/Nck^H^ strain (Table 2): seven mutations augmented or accelerated the development of T1D (Figs 2 and 3), and five mutations suppressed or delayed the development of T1D (Fig. 4). Further studies are necessary to validate candidate mutations in mice with independently generated mutant alleles on a clean NOD/Nck^H^ background.

**Table 2.**
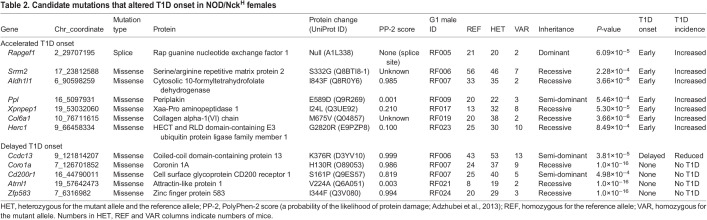
Candidate mutations that altered T1D onset in NOD/Nck^H^ females

Because large cohorts of female mice were produced for analysis, it was possible to have groups of mice within the same pedigree in which particular mutations were exclusively present (or nearly so). For example, in RF006, two mutations (in *Ccdc13* and *Srrm2*) modified in opposite directions the age of T1D onset in NOD/Nck^H^ mice. Excluding seven mice homozygous for the recessive *Srrm2* mutation, we analyzed the remaining 102 mice, among which were 12 mice homozygous for the *Ccdc13* mutation, 51 heterozygotes and 39 WT; we observed association of homozygosity or heterozygosity for the *Ccdc13* mutation with delayed T1D onset independent of *Srrm2* genotype. Thus, one advantage of our approach is that we could perform single locus linkage analysis independently for multiple mutations within a single pedigree. We observed two candidate loci in pedigree RF006, and three in RF007, that independently altered T1D onset age (Table 2). Another important feature of the approach is the ability to detect semi-dominant effects, as observed for mutations in *Ppl* (Fig. 2G,H), *Ccdc13* (Fig. 4A,B) and *Cd200r1* (Fig. 4E,F); considering that an individual human may have ∼10,000 non-synonymous SNPs of which ∼50% are heterozygous (Ng et al., 2008; Wheeler et al., 2008), detecting heterozygous mutation effects in disease models will likely have clinical relevance.

## DISCUSSION

In this work, we present a forward genetic screen for mutations that modify the age of T1D onset in NOD/Nck^H^ mice, a high T1D incidence subline (Foray et al., 2021). In all cases of accelerated phenotype, we found that an earlier onset was associated with greater incidence of T1D. Conversely, phenotypes in which T1D was delayed were associated with reduced incidence of T1D (in four out of five cases, completely suppressed). Among the 12 candidate genes, the involvement of 11 of them in autoimmune disease, T1D or β-cell homeostasis had not previously been reported. Based on a genome saturation of 0.35% with damaging or null alleles, and the identification of five suppressing and seven exacerbating mutations, we estimate a genomic footprint of approximately 1400 and 2000 total genes, respectively, within which loss-of-function mutations might be expected to delay/suppress or accelerate/augment T1D in NOD/Nck^H^ female mice. These are not unreasonable estimates given that most known T1D-associated loci have small effect sizes (odds ratio <1.5), with the exception of HLA class II haplotypes, which account for ∼40% of the risk for T1D (Pociot, 2017; Robertson et al., 2021); thus, many, perhaps thousands, of gene variants may contribute to disease. Conversely, many gene variants may protect against disease. It appears that a fine balance between numerous predisposing and protective variants determines disease outcome with respect to T1D.

As in human T1D, an autoimmune etiology causes T1D in NOD mouse strains. Destruction of insulin-secreting β cells in T1D is dependent upon a tight cooperation between CD4^+^ and CD8^+^ autoreactive T lymphocytes. Although CD4^+^ T cells are essential for the triggering and progression of diabetes, T1D development requires important contributions from cytotoxic CD8^+^ T cells that destroy β cells of the islets of Langerhans, leading to insufficiency of circulating insulin in NOD mice (Lieberman et al., 2003; Verdaguer et al., 1997; Trudeau et al., 2003). Consistent with this understanding and validating the effectiveness of the screen, we found that a putative damaging missense mutation of *Coro1a* (H130R) protected 100% of NOD/Nck^H^ female mice from T1D. Coronin 1A has an established role in supporting T-cell trafficking and survival (Föger et al., 2006; Mueller et al., 2008; Shiow et al., 2008), and flow cytometric studies confirmed marked deficiency of circulating T cells (expressing high CD44 levels) in *Coro1a^H130R/H130R^* mice (Fig. S2) in accordance with the observed suppression of T1D.

The loss of self-tolerance leading to T1D in NOD mice has been substantiated by the identification of at least 18 islet antigens targeted by CD4^+^ and CD8^+^ T cells, including insulin, glutamate glutamic acid decarboxylase (GAD), the protein tyrosine phosphatase-like IA-2, islet-specific glucose-6-phosphatase catalytic subunit-related protein (IGPR) and heat shock protein 60 (HSP60) (Babad et al., 2010; James et al., 2020). Diabetogenic T cells may arise and persist because of defects in both central and peripheral tolerance, resulting in elevated diabetes susceptibility (Anderson and Bluestone, 2005; Bach, 1994). For example, abnormalities in thymic selection and development in NOD mice have been reported that lead to impaired central tolerance; in addition, changes in Th1 versus Th2 responses, co-stimulation, and regulatory T cell (Treg) function reduce peripheral tolerance in NOD mice (reviewed by Anderson and Bluestone, 2005; Bach, 2003). We speculate that candidate mutations in *Herc1*, *Xpnpep1* and *Srrm2*, associated with earlier onset and increased T1D incidence, may alter the MHC peptidome in thymic epithelial cells, thereby favoring the survival of autoantigen-specific T cells during thymic selection. All three mutations are recessive, suggesting that loss of function of the encoded proteins is responsible for the accelerated T1D phenotype. *Herc1* encodes the large (4859 aa) HECT and RLD domain-containing E3 ubiquitin protein ligase family member 1, one of two members of the large HERC family present in most metazoans (García-Cano et al., 2019). In mice, HERC1 is widely expressed with highest transcript expression in the brain (Bastian et al., 2021), and a spontaneous missense mutation (G483E) resulted in delayed growth, short body, high juvenile mortality and severe ataxia as a result of Purkinje cell degeneration in mice (Mashimo et al., 2009). XPNPEP1 is a cytosolic aminopeptidase P believed to have broad substrate specificity (Harbeck and Mentlein, 1991). XPNPEP1 cleaves the N-terminal residue of proteins and peptides containing an adjacent proline (i.e. a proline at the second position) (Cottrell et al., 2000; Erşahin et al., 2005), which may lead to degradation of the substrate (Li et al., 2008; Cottrell et al., 2000; Erşahin and Simmons, 1997). Mice homozygous for a gene trap mutation in *Xpnpep1* exhibited pre- and postnatal lethality, reduced male survival, growth retardation, microcephaly, peptiduria, behavioral hyperactivity, epileptic EEG discharges, and impaired hippocampus-dependent learning and memory (Yoon et al., 2012; Bae et al., 2018). SRRM2, also known as SRm300, is a spliceosome component that functions as a regulatory factor in the catalytic center of the spliceosome C complex after the first catalytic step (Grainger et al., 2009; Khanna et al., 2009). Loss of function of the ortholog of SRRM2 is non-lethal in yeast and results in accumulation of unspliced transcripts (Blencowe et al., 2000; Grainger et al., 2009; Khanna et al., 2009), whereas in mice *Srrm2* knockout is lethal [http://www.informatics.jax.org/allele/MGI:6257609 (accessed 12/14/2021)]. By altering the set of proteins degraded (*Herc1* and *Xpnpep1* mutations) or synthesized (*Srrm2* mutation), these candidate mutations may all conceivably alter the immunopeptidome presented by MHC class I and/or MHC class II and hinder negative selection of islet protein-specific T cells.

Multiple antigens may be capable of initiating and/or contributing to T1D pathogenesis, including hybrid antigens produced in β cells and consisting of covalent fusions between insulin fragments and peptides derived from secretory granule proteins such as chromogranin A and IAPP (Delong et al., 2016; Wiles et al., 2017). Mutations in islet proteins themselves, by changing the affinity of peptide(s) containing them for either MHC class I or MHC class II, may enhance or diminish MHC presentation of these autoantigens to affect T1D susceptibility. Periplakin (encoded by *Ppl*) and collagen α-1 (VI) chain (encoded by *Col6a1*), are extracellular matrix/cytoskeleton components expressed in the pancreas in addition to the skin (Kazerounian et al., 2002; Li et al., 2021). The implication of two mutations affecting such extracellular matrix/cytoskeleton proteins lends weight to their possible association with accelerated T1D development. Notably, the missense mutation E589D in PPL is predicted to be benign (Adzhubei et al., 2013), suggesting that a functional deficit may not be responsible for the acceleration of disease in our screen. Because NOD/Nck^H^ mice develop T1D in the absence of mutations in either *Ppl* or *Col6a1*, it is unlikely that these mutations create disease-inciting autoantigens. Rather, once initiated the pathogenic disease process may involve epitope spreading to PPL and COL6A1 antigens, favored by potentially enhanced binding of mutant peptides to MHC molecules.

Although an adaptive immune response to β cells is essential for T1D to develop in NOD mice, as evidenced by complete absence of T1D in mice lacking T cells, it is not necessarily sufficient. It is an open question whether NOD mice are differentially sensitive to an immune response, perhaps at the β-cell level, once it has begun. Thus, compelling evidence supports the idea that β cells resist the assault of the pathogenic immune response through an intricate cross-talk of soluble mediators and membrane receptors resulting in fine-tuning of anti- and pro-apoptotic genes (Santin and Eizirik, 2013; Størling and Pociot, 2017). We identified a mutation in *Aldh1l1*, a pancreas-enriched gene (Krupenko et al., 2010), that accelerated the development of T1D in NOD/Nck^H^ females. The mutation (I843F) is predicted to be damaging to protein function (Adzhubei et al., 2013). The gene is reportedly not expressed in leukocytes, suggesting that it may function in pancreatic cells to protect against immune attack.

Hypotheses can be made concerning the effects of mutations in the genes encoding RAPGEF1 (accelerated T1D) and ZFP583, CCDC13, CD200R1 and ATRNL1 (delayed T1D), but are exceedingly speculative because known functions of these proteins (if any) do not fit neatly within the current framework for T1D etiology. For example, a PubMed search for the predicted transcription repressor ZFP583 retrieved zero results, but a mouse β cell-specific knockout of the chromatin remodeling Swi/Snf proteins BRG1 (SMARCA4) and BRM (SMARCA2) resulted in pancreas hypoplasia, glucose intolerance, hyperglycemia and impaired insulin secretion; in these mice, β-cell *Zfp583* gene expression was upregulated 2.75-fold over the WT expression level (Spaeth et al., 2019). Could reduced ZFP583 function caused by a damaging missense mutation (I344F) confer a T1D protective effect? An interesting link has been reported between type 2 diabetes (T2D) and CCDC13, a centriolar satellite protein required for ciliogenesis and genome stability (Staples et al., 2014); recent work suggests that primary cilia are important for islet β-cell glucose sensing, calcium influx, insulin secretion, and cross regulation of α and δ cells (Hughes et al., 2020). However, our data indicate a protective effect of a putative damaging mutation in *Ccdc13* on T1D. There is also a T2D association with variants of human *RAPGEF1* by GWAS in Chinese, Finish and Korean populations (Gaulton et al., 2008; Hong et al., 2009; Qu et al., 2011), suggesting that precocious manifestation of diabetes in NOD/Nck^H^ mice with putative null *Rapgef1* mutation may result from peripheral insulin resistance combined with failing insulin production. Overall, with the exception of a coronin 1A mutation, our screen implicated 11 mutations in proteins outside of canonical immunoregulatory pathways or known autoantigens in NOD mice, suggesting that novel aspects of T1D pathogenesis await discovery.

To our knowledge, a forward genetic modifier screen on the NOD background has not previously been attempted. A major advantage of this approach over QTL mapping using congenic NOD mice is that mutagenesis coupled with AMM almost always permits unambiguous identification of single gene variants responsible for phenotypic effects. The complex nature of disease in the NOD strain is preserved and single gene effects can be interrogated one at a time without the need for extensive backcrossing to isolate QTLs. We expect that the genes and variants identified in screening will increase our understanding of disease pathways and pathogenesis in human T1D, leading to identification of targets for the treatment of T1D.

## MATERIALS AND METHODS

### Mice

NOD/Nck^H^ and NOD/Nck^L^ are two sublines established from the NOD/Nck substrain bred in our laboratory since 1986 (Foray et al., 2021; Hunger et al., 1996) from the original Japanese NOD/ShiJcl colony (Central Laboratory of Experimental Animals, Japan). NOD/Nck^H^ (high incidence) and NOD/Nck^L^ (low incidence) mice differed in T1D incidence, a phenotypic variation that was stably maintained for several generations (Foray et al., 2021). NOD/Nck.*Rag1^−/−^* mice were generated by crossing NOD/Nck *Rag1^−/−^* mice (raised in our laboratory) and NOD/Nck^H^ mice. Mice were bred and housed under specific pathogen-free conditions at the Hôpital Necker-Enfants Malades animal facility (agreement: C751515). Animals were fed *ad libitum* with an irradiated VRF1 diet (Special Diets Services) with fresh autoclaved water.

This study was carried out in strict accordance with the recommendations of European Directives (2010/63/UE) and institutional guidelines (INSERM, Université Paris Cité). The protocols were approved by the Ethical Committee of Paris Descartes University and the French Ministry of Education and Research.

### ENU treatment

Twenty-one 4-week-old NOD/Nck^L^ and 18 4-week-old NOD/Nck^H^ male mice were used (weight ranging 20-25 g). Mice were randomly assigned to two groups receiving 100 mg/kg ENU (ten NOD/Nck^L^ and eight NOD/Nck^H^) or 125 mg/kg ENU (11 NOD/Nck^L^ and eight NOD/Nck^H^). ENU powder (Sigma-Aldrich) was dissolved in phosphate buffer (pH 6.0) to 2 mg/ml and used immediately after solubilization. ENU was injected at each dose intraperitoneally once a week for three consecutive weeks. A single NOD/Nck^L^-treated mouse was euthanized each week from 1 to 9 weeks after the third ENU injection to analyze spermatogenesis (testis histology and sperm count from cauda epididymis). Three NOD/Nck^H^ males treated with the lower dose of ENU survived and regained fertility and were bred to produce three generations of mice (G1, G2, G3; see Fig. 1A). G1 male pedigree founders were exome sequenced. Female G2 and G3 mice were screened for phenotypes by diabetes monitoring.

### Sperm counts

Cauda epididymides were minced and homogenized on ice in 1.0 ml of physiological saline solution with iris scissors until no obvious piece of tissue was visible by eye. The homogenate was filtered, and then 10 µl of sample were diluted 1:10 in saline buffer containing 4% Trypan Blue (vol/vol) and counted under a phase contrast microscope at 200× magnification using a hemocytometer.

### Histological analysis

For light microscopy, testicles were fixed in formaldehyde solution [e.g. Sigma-Aldrich, 252549 containing 37% formaldehyde in water (wt/wt) (85%), mixed with ethanol (10%) and glacial acetic acid (5%) at the indicated vol/vol percentages] for 24 h, subjected to routine processing for paraffin-embedded tissue samples, sectioned at 5 µm and stained with Hematoxylin and Eosin.

### Diabetes monitoring

Mice were monitored for diabetes weekly by testing for glycosuria using colorimetric Diabur-Test 5000 strips (Roche). Overt diabetes was confirmed by testing for fasting glycemia >250 mg/dl (Accu-Check; Roche).

### Genomic DNA extraction from whole blood and exome sequencing

Genomic DNA was extracted from fresh whole blood (100-150 µl) of G1 male founders using RBC Lysis Solution (Qiagen, 158902) according to the instructions of the manufacturer. A NanoDrop spectrophotometer (Thermo Fisher) was used to determine gDNA concentration (total yield 2-20 µg gDNA). Whole-exome sequencing was performed as previously described (Wang et al., 2015) except that variants relative to the published NOD reference sequence (Steward et al., 2013) with quality scores ≥40 were annotated as potential mutations.

### AMM

AMM was performed as previously described (Wang et al., 2015). Briefly, genomic DNA was extracted from tail snips of G2 and G3 female mice and genotyped at the mutation sites identified in their related G1 founders. Custom primers (Paragon Genomics) were used to amplify each locus followed by sequencing using an Ion PGM (Life Technologies). Following phenotypic screening, linkage analysis using recessive, semi-dominant (additive) and dominant models of inheritance was performed for every mutation in the pedigree using the program Linkage Analyzer (Wang et al., 2015). Kaplan–Meier plots and Manhattan plots were generated using the program Linkage Explorer. The *P*-values of association between genotype and phenotype were calculated based on Kaplan–Meier analysis of time of onset of T1D, as related to zygosity for each of the mutations using a likelihood ratio test from a generalized linear model or generalized linear mixed effect model and Bonferroni correction applied. Note that because all G3 females (from multiple G2 mothers that may be heterozygous or WT for the mutation) were screened in one large group together with multiple G2 females that had the same G1 father, the numbers of mice of each genotype in Figs 2-4 are not expected to conform to Mendelian ratios.

### Flow cytometry

Thirty to fifty microliters of whole blood were collected into heparin-containing Eppendorf tubes. Red blood cells were lysed (L3289, Sigma-Aldrich lysis solution) and sample washed. Surface staining was performed at 4°C in staining buffer (2% fetal calf serum and 5 mM EDTA in PBS) using fluorochrome-labeled antibodies to murine CD45 (30-F11), CD19 (1D3), CD3 (145-2C11), CD4 (GK1.5), CD8 (53-6.7) and CD44 (KM114), which have been validated for flow cytometry by the manufacturer (BD Pharmingen). Data were acquired on a FACSFortessa flow cytometer (BD Biosciences) and analyzed using FlowJo version 10 software (Tree Star).

### Methodology and statistical analyses

Phenotypic screening was restricted to female mice because they showed a higher initial incidence of T1D than male mice. This pre-established criterion was based on the hypothesis that more suppressor mutations might be identified in a group with high initial T1D frequency. The investigator was blind to genotype during diabetes monitoring. For incidences, statistical analyses were performed using GraphPad Prism version 6 software. Cumulative actuarial diabetes incidence was calculated according to the Kaplan–Meier method. Incidence curves were compared using the logrank (Mantel–Cox) test.
